# Regulatory Roles of Invariant Natural Killer T Cells in Adipose Tissue Inflammation: Defenders Against Obesity-Induced Metabolic Complications

**DOI:** 10.3389/fimmu.2018.01311

**Published:** 2018-06-11

**Authors:** Yoon Jeong Park, Jeu Park, Jin Young Huh, Injae Hwang, Sung Sik Choe, Jae Bum Kim

**Affiliations:** ^1^Department of Biological Sciences, Center for Adipose Tissue Remodeling, College of Natural Sciences, Institute of Molecular Biology and Genetics, Seoul National University, Seoul, South Korea; ^2^Department of Biophysics and Chemical Biology, Seoul National University, Seoul, South Korea; ^3^Department of Medicine, University of California San Diego, San Diego, CA, United States

**Keywords:** adipocytes, invariant natural killer T cells, obesity, inflammation, CD1d

## Abstract

Adipose tissue is a metabolic organ that plays a central role in controlling systemic energy homeostasis. Compelling evidence indicates that immune system is closely linked to healthy physiologic functions and pathologic dysfunction of adipose tissue. In obesity, the accumulation of pro-inflammatory responses in adipose tissue subsequently leads to dysfunction of adipose tissue as well as whole body energy homeostasis. Simultaneously, adipose tissue also activates anti-inflammatory responses in an effort to reduce the unfavorable effects of pro-inflammation. Notably, the interplay between adipocytes and resident invariant natural killer T (iNKT) cells is a major component of defensive mechanisms of adipose tissue. iNKT cells are leukocytes that recognize lipids loaded on CD1d as antigens, whereas most other immune cells are activated by peptide antigens. In adipose tissue, adipocytes directly interact with iNKT cells by presenting lipid antigens and stimulate iNKT cell activation to alleviate pro-inflammation. In this review, we provide an overview of the molecular and cellular determinants of obesity-induced adipose tissue inflammation. Specifically, we focus on the roles of iNKT cell-adipocyte interaction in maintaining adipose tissue homeostasis as well as the consequent modulation in systemic energy metabolism. We also briefly discuss future research directions regarding the interplay between adipocytes and adipose iNKT cells in adipose tissue inflammation.

## Introduction

White adipose tissue (WAT) is a central controller of lipid and glucose homeostasis that communicates locally and with distant tissues. The mass of WAT expands or reduces dynamically in response to nutritional states. WAT actively senses nutritional changes and accordingly stores extra energy in the form of triglycerides or supplies nutrients to other organs (1, 2). Generally, WAT expands both by hyperplasia (an increase in mature adipocyte number) and hypertrophy (an increase in mature adipocyte size) (3, 4). White adipocytes are the major cell type of WAT and normally contain a single large lipid droplet. White adipocytes crosstalk with multiple cell types in both local and remote tissues *via* the secretion of a variety of signaling molecules (1, 2).

Traditionally, the immune system has been considered central to the elimination of pathogenic microbes and toxic or allergenic molecules that threaten the normal homeostasis of the host. A more recent addition to the broad discussion of immunity in health and diseases is the role of the interplay between immune response and metabolism (5). In particular, the roles of this interplay in obesity and metabolic diseases have been suggested by the findings that the immune program is intimately linked to physiological and pathological changes in WAT (6–9). One example is the inappropriately active and/or overactive immune responses in WAT in obesity and its related metabolic diseases. Along with enhanced WAT expansion, obesity induces both quantitative and qualitative changes in WAT immunity, which potentiates the dysfunction of adipose tissue as well as systemic energy homeostasis (10–12).

Among the resident immune cells in WAT, invariant natural killer T (iNKT) cells are regarded as one of the key players linking dynamic changes in adipocyte metabolisms to WAT homeostasis (13). In the following review, we briefly discuss different molecular and cellular factors involved in the control of WAT immunity in obesity. In particular, we emphasize the roles of the interaction between iNKT cells and adipocytes in maintaining WAT homeostasis as well as whole body energy metabolism.

## WAT Immunity in Obesity

Obesity is defined as the massive expansion of WAT due to the imbalance between caloric intake and energy expenditure. In adult obesity, WAT expansion features by dramatic increases in the number of hypertrophic adipocytes that are significantly related to detrimental changes in WAT, including hypoxia, oxidative stress, and insulin resistance (3, 4). Obesity is strongly associated with interrelated metabolic diseases, including insulin resistance, type 2 diabetes, and cardiovascular disease, which impose a high social burden in terms of quality of life (3, 4). Given that WAT is the major organ for energy storage and mobilization, most previous obesity-related studies focused on finding abnormalities in adipocyte physiology and metabolism in effort to understand the link between obesity and metabolic diseases (14). However, the recent discovery of adipokines, an array of mediators secreted by adipose tissue, has revised the concept of WAT being merely a fat storage depot (3, 4). Instead, it has become clear that WAT is a dynamic endocrine system that is crucial in the regulation of systemic energy homeostasis.

Adipokines include angiogenic proteins, metabolic regulators, and inflammatory mediators. Most adipokines including leptin and adiponectin act as the bridge between the functional status of WAT and other organs, modulating systemic energy metabolism (3, 4). Among various adipokines, the identification of inflammatory mediators has clarified the connection between immunity and obesity and its related metabolic diseases (15). The first study that established the reframing of obesity as an inflammatory condition demonstrated the detrimental effect of tumor necrosis factor alpha (TNF-α), an inflammatory mediator secreted by adipose tissues, on insulin resistance in many animal models of obesity (16). Subsequent studies enforced the idea that alterations in WAT immunity are closely associated with dynamic changes in energy homeostasis in obesity and metabolic diseases (8, 9).

One hallmark characteristic of WAT immunity in obesity is chronic low-grade inflammation, which leads to a modest increase in circulating pro-inflammatory factors (8, 9). In a lean state, WAT immunity is skewed toward the anti-inflammatory phenotype, which supports tissue expansion (3, 4). In obesity, nutritional stresses promote the secretion of inflammatory cytokines and acute-phase reactants including TNF-α, interleukin (IL)-6, and serum amyloid A in WAT. Although WAT simultaneously increases the release of anti-inflammatory cytokines, including IL-4, IL-10, and IL-2 to counteract the unfavorable effects of inflammation, WAT immunity eventually shifts toward an inflammatory state, leading to prolonged inflammation in obesity (8, 9, 13).

## Key Players of WAT Inflammation in Obesity

White adipose tissue is a heterogeneous organ composed of white adipocytes, mural endothelial cells, fibroblasts, and various immune cells, including macrophages, T cells, B cells, and NKT cells. These cells are engaged in maintaining the well-being of adipocytes, clearance of apoptotic cells, and retaining healthy physiological functions of WAT. Particularly, obesity-induced multiple insults, including epigenetic malfunction, hypoxia, and oxidative stress have complex impacts on adipose tissue inflammation by altering inter-cellular interaction between adipocytes and immune cells. For instance, recent studies have underscored the important roles of epigenetic modulators in the progression of adipokine dysregulation and subsequent adipose tissue inflammation (17, 18). In obese WAT, hypoxia and oxidative stress work in concert to promote dysfunction of adipocytes and lead to the stimulation of inflammatory signaling pathways in neighboring immune cells (19–22).

**Figure 1:**
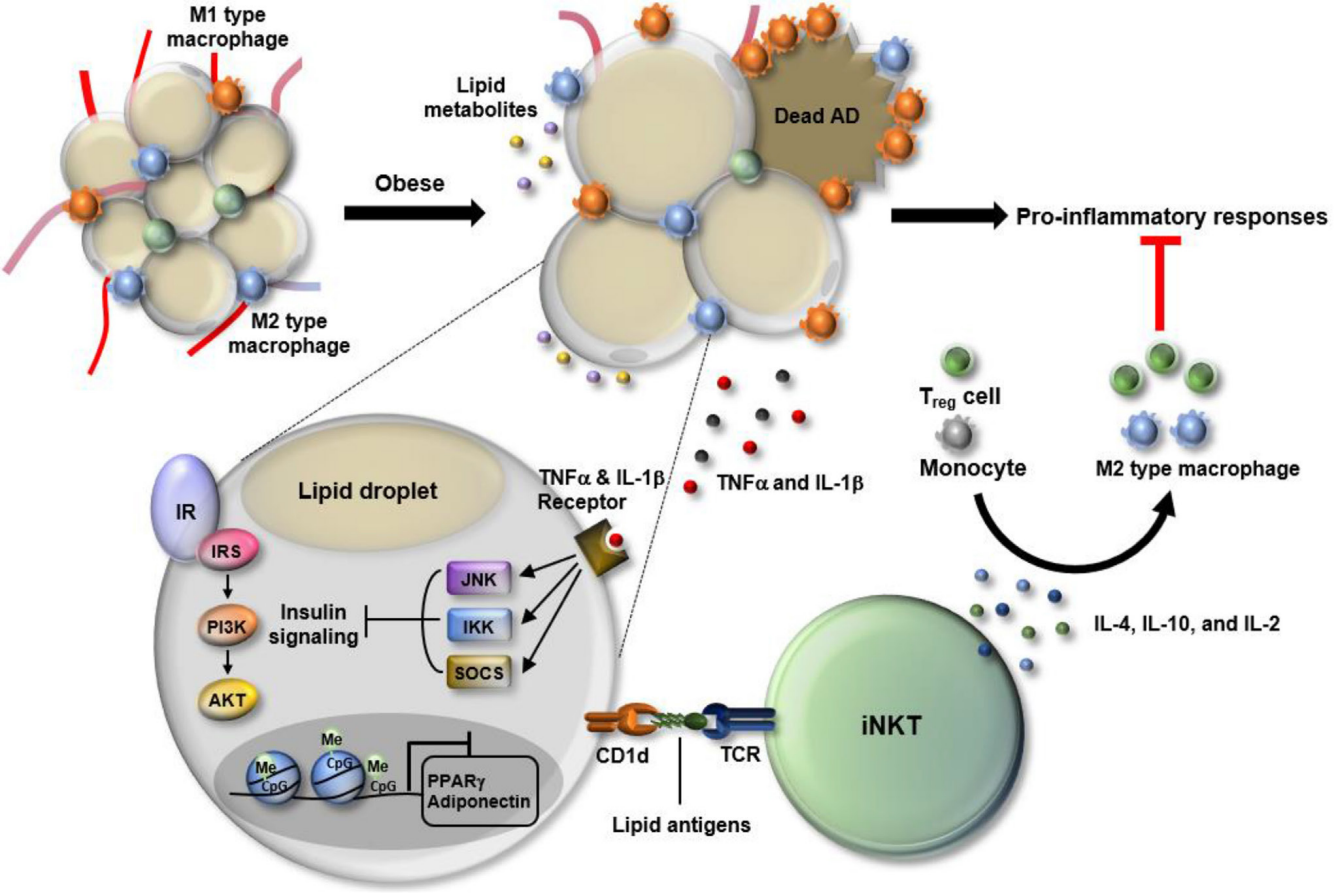
Dynamic changes in white adipose tissue (WAT) immunity in obesity. In the progression of obesity, WAT faces multiple stresses, including hypoxia, oxidative stress, and epigenetic malfunction. Particularly, adipocytes become enlarged in the process of absorbing excess nutrients, which is accompanied by adipocyte death and leakage of lipid metabolites. Also, obesity-induced DNA hypermethylation in adipocytes leads to the suppression of genes involved in adipocyte function including peroxisome proliferator-activated receptor gamma (PPARγ) and adiponectin. In response to such changes, WAT immunity skews toward pro-inflammatory state. Among cells residing in WAT, M1-type macrophages secrete a variety of cytokines, such as TNF-α and interleukin (IL)-1β that activate JNK, IKK, and SOCS, leading to suppression of insulin signaling in WAT. Adipose invariant natural killer T (iNKT) cells have anti-inflammatory roles as a part of defense mechanism to resolve pro-inflammatory responses. Adipose iNKT cells are mainly activated by lipid antigens loaded onto adipocyte CD1d and secrete Th2-type cytokines, such as IL-4, IL10, and IL-2. Those cytokines drive the polarization of monocytes toward M2 type macrophages and activate regulatory T cells, contributing to an alleviation of the pro-inflammatory responses in WAT.

Among various cells in WAT, adipocytes act as both sensors and messengers that form an early warning network of WAT immunity (Figure 1). In response to excessive nutritional overload, adipocytes undergo both metabolic and immunologic reprogramming, which includes dramatic changes in metabolite and lipid compositions (23, 24). Following reprogramming, adipocytes alert neighboring immune cells to eliminate such stresses through the secretion of an array of cytokines and presentation of certain types of antigens that reflect dynamic alterations in WAT under stressful conditions (25–28).

Macrophages are another key player in adipose tissue inflammation. These cells are the primary source of pro-inflammatory cytokines in obese WAT (8, 9). Monocytes differentiate into classically activated macrophages (M1) or alternatively activated macrophages (M2) according to the stimuli. M1 macrophages are pro-inflammatory, whereas M2 macrophages are anti-inflammatory (29). The balance between M1 and M2 macrophages is crucial to maintain WAT homeostasis, and the disturbance in this balance triggers the pathologic dysfunction of WAT (Figure 1). In obesity, the proportion of M1 macrophages is significantly increased compared to M2 macrophages, which confers a vicious cycle of WAT inflammation through having multiple impacts on other cells (29–31).

Simultaneously, WAT also promotes anti-inflammatory responses in effort to alleviate pathologic dysfunction of adipose tissue (8, 9). In WAT, there are many types of cells involved in anti-inflammatory responses, including M2 macrophages, eosinophils, regulatory T (Treg) cells, and iNKT cells. Depletion of anti-inflammatory cells in animal models of obesity accelerates WAT inflammation and consequently aggravates metabolic disorders including insulin resistance (26, 32–36). These studies suggest that, despite of a dominant role of pro-inflammatory response, anti-inflammatory response is still required to dampen WAT inflammation.

## Distinct Characteristics of NKT Cells

NKT cells are innate-like T lymphocytes that function similarly to innate cells, displaying less specificity and more rapid activation compared to adaptive immune cells (37, 38). NKT cells can be activated by exogenous or endogenous lipid antigens, and by cytokines produced by antigen-presenting cells (APCs). Upon activation, NKT cells rapidly secrete a variety of cytokines. Moreover, NKT cells express cytotoxic granules containing perforin and granzyme, and induce apoptosis of target cells (37). NKT cells are largely categorized into three types: iNKT cells (type I), diverse NKT cells (dNKT, type II), and NKT-like cells (37). Although both type I and type II NKT cells recognize lipid antigens loaded on the MHC class I-like family protein CD1d, they are activated by distinct types of lipid antigens *via* the expression of different repertoires of T-cell receptors (37, 38). For instance, iNKT cells express a conserved semi-invariant TCR and are potently stimulated by α-galactosylceramide (α-GC), a marine sponge-derived glycolipid (39). Type II NKT cells express a broader TCR repertoire and sulfatide is one of the major antigens for these cells (40, 41). Among subsets of the NKT cell population, iNKT cells have been suggested to modulate WAT immunity in both lean and obese individuals (25, 26, 34, 35, 42). One of the interesting features of iNKT cells is their remarkable functional plasticity with both pro- and anti-inflammatory characteristics. Upon activation signaling, iNKT cells secret either robust Th1-type or Th2-type cytokines according to the nature of activating stimuli and types of APCs and cytokines (43).

## Adipocytes as Profound APCs for iNKT Cells

While conventional naïve T cells are mostly localized to immune organs, iNKT cells reside in many tissues, with a relatively high abundance in the liver and WAT (25, 44). Along with iNKT cells, such tissues appear to have distinct pools of APCs that rapidly process and present lipid antigen to confer tissue-specific function to iNKT cells. Generally, dendritic cells, macrophages, and B cells are considered “professional” APCs as they express various components required for lipid antigen synthesis and presentation. They function as the major APCs that modulate the differentiation and activity of iNKT cells in lymphatic organs, including the thymus and spleen (38, 43). “Non-professional” APCs are not conventional APCs, but express CD1d. Interestingly, iNKT cells residing in metabolic tissues are promptly activated by non-professional APCs (25, 44). Among the APCs in WAT, adipocytes seem to be a efficient non-professional APCs with the highest levels of CD1d in parallel with expression of other factors required for lipid antigen presentation (13, 25). As obesity is closely associated with major changes in the lipid repertoire of adipocytes, considerable attention has been directed to the role of adipocyte-derived lipids in the control of WAT inflammation (45–47). Owing to the primary function of adipocytes being endocrine cells, most studies related to obesity have focused on the link between the immune system and secreted lipids (45, 46). However, given that adipocytes express the highest level of CD1d among the resident APCs in WAT and since lipid metabolites can act as “antigenic” lipids after being loaded on CD1d, it is very likely that endogenous lipid metabolites derived from adipocytes may act as antigenic lipids (13, 25). Indeed, adipocyte cell line and primary adipocytes isolated from both mouse and human activate iNKT cells and stimulate cytokine secretion (26, 35). Moreover, adipocytes are able to promote cytokine secretion of iNKT cells without exogenous lipid antigens, such as α-GC, implying the presence of adipocyte-derived lipid antigens (28, 35). Very recently, we and other groups have reported the *in vivo* role of adipocytes as APCs by the use of an adipocyte-specific CD1d knockout (CD1d^ADKO^) mouse model (25, 28). In these studies, lean CD1d^ADKO^ mice exhibit reduced number of iNKT cells in WAT and have different cytokine profiles in adipose iNKT cells compared to control mice in obesity (25).

In nature, iNKT cells recognize a vast range of lipid antigens, which includes microbial lipids and self-lipid antigens. Generally, such lipid antigens are composed of sugar moieties linked to a lipid backbone that can either be based on a ceramide or a diacylglycerol (48–51). Recently, several reports demonstrated that obesity induces the activation of enzymes involved in ceramide synthesis in conjunction with the elevation of cellular ceramides in mouse and human WAT (52, 53). Ceramide is found in high concentrations within cell membranes and is used as a precursor molecule for the synthesis of glycolipids. Given that many self-lipid antigens contain ceramide backbones and that these antigens are more potent than antigens based on diacylglycerol, an increase in ceramide-mediated glycolipids might contribute to the enrichment of lipid antigen pools in adipocytes as well as subsequent activation of adipose iNKT cells (54). However, the structural basis of adipocyte-derived antigens including types of lipid backbone and sugar moieties has not been fully explored and is a promising avenue of investigation. Also, further analyses of the effects of nutrient stresses on characteristics of adipocyte-derived lipid antigens that modulate expansion of iNKT cell population and specific Th1 or Th2 cytokine profiles could have promising therapeutic potential concerning WAT inflammation.

## Adipose iNKT Cells and their Roles in WAT Inflammation

White adipose tissue harbors a distinct pool of cells of the immune system (Figure 1). The characteristics of the cells are often governed by adipose tissue-specific cues including antigens. In lean WAT, adipose iNKT cells account for 1–20% of the resident T-cell pool (34). The majority of adipose iNKT cells are tissue-resident lymphocytes, whereas a small portion of the cells is infiltrated into WAT (42). Adipose iNKT cells produce anti-inflammatory cytokines, such as IL-4 and IL-10, and regulate the function of M2 macrophages and Treg cells, which contribute to the maintenance of WAT homeostasis (Figure 1). Recent reports described several characteristics unique to adipose iNKT cells. The transcriptome of adipose iNKT cells differs from the transcriptome of iNKT cells residing in other tissues (42). Among surface markers defining iNKT cells, the expression of CD4 and NK1.1 is relatively low in adipose iNKT cells (42). Also, adipose iNKT cells are less dependent on promyelocytic leukemia zinc finger (PLZF), a key transcription factor responsible for iNKT activation (42). Adipose iNKT cells express little PLZF compared to other iNKT cells and the quantity of adipose iNKT cells is not affected in *Plzf*^+/−^ mice (42). Instead, the levels of T-bet, GATA-3, and E4BP4 are high in adipose iNKT cells (42). Finally, adipose iNKT cells seem to be chronically activated, while iNKT cells in the rest of body are in a poised state that requires an additional signal for rapid cytokine production (42). Such a distinct activation state of adipose iNKT cells results from special microenvironments, including lipid antigens, cytokines, and adipokines in WAT.

In obesity, WAT undergoes dramatic changes in the immune system favoring a pro-inflammatory environment. Notably, the number of adipose iNKT cells significantly declines in parallel with elevation of inflammation in WAT (26, 34, 55). Recent reports from several groups including ours have shown that iNKT-cell-deficient mouse models (*J*α*18^−/−^* and *CD1d^−/−^* mice, which are deficient in iNKT cells and both iNKT cells and type II NKT cell, respectively) are more susceptible to obesity, adipose tissue inflammation, as well as insulin resistance on a high-fat diet regimen (26, 34). These phenotypes are reversed by the adoptive transfer of iNKT cells or specific activation of iNKT cells with α-GC, supporting the protective role of iNKT cells in obesity (26, 34). Furthermore, the impaired induction of arginase-1, an M2 macrophage marker gene, was reported in *CD1d^−/−^* mice (33). Collectively, these studies reveal that adipose iNKT cells are crucial to maintain WAT homeostasis due to their ability to secrete anti-inflammatory cytokines. Thus, the dramatic decrease in adipose iNKT would constitute an important initiator of the microenvironment favorable for inflammation in WAT. However, whether adipose iNKT cells act only as anti-inflammatory cells in obesity is debatable. Other studies suggest that iNKT cell deficiency leads to a decrease in obesity-induced adipose tissue inflammation and insulin resistance (56, 57). For instance, Satoh et al. demonstrated that CD1d^ADKO^ mice exhibit improved insulin sensitivity and adipose tissue inflammation (28). There are also other reports suggesting that iNKT cells are dispensable for adipose tissue inflammation as well as systemic energy homeostasis (58, 59). Multiple factors could be attributable to such a difference in adipose iNKT polarization in relation to obesity and adipose tissue inflammation. These factors include the type of diet, duration of diet intervention, types of control mouse groups, and gut microbiota (25, 26, 28, 34, 56). Particularly, the potential effect of gut microbiota on adipose iNKT cells appears to be interesting. A very recent study demonstrated that glucagon-like peptide-1 (GLP-1), a gut hormone that is used to treat obesity and diabetes, activates adipose iNKT cells and enhances the secretion of anti-inflammatory cytokines including IL-10 in WAT (60). Like other gut hormones, serum GLP-1 level is sensitively modulated by the composition of gut microbiota in response to changes in the nutritional status (61). Thus, it is probable that different composition of gut microbiota among different laboratories would impact on GLP-1-adipose iNKT axis, accounting for the differences in the function of adipose iNKT cells that have been reported in obesity.

## Future Research Directions in Adipose iNKT Cells

**Figure 2:**
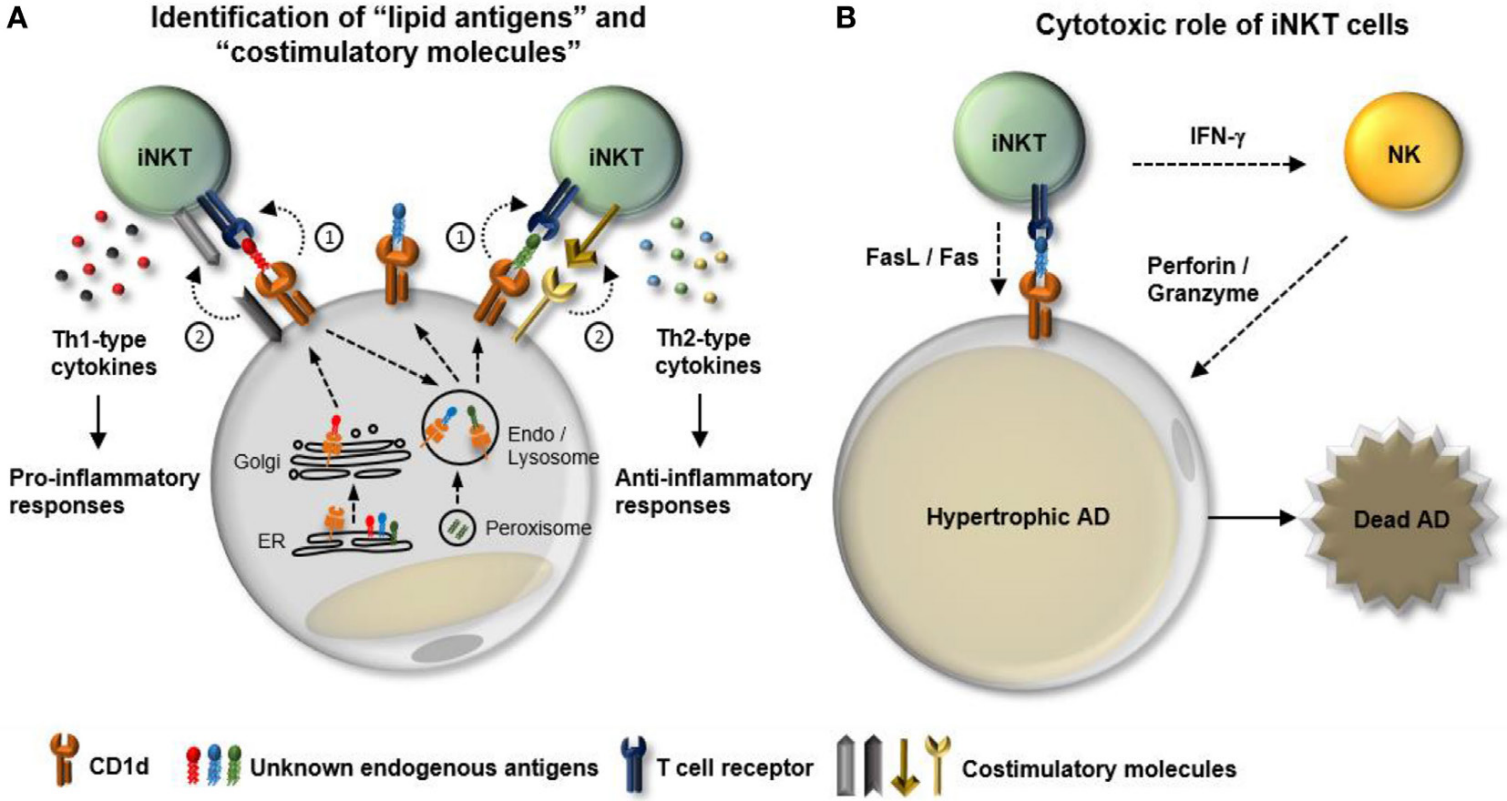
Potential mechanisms involved in the interplay between adipocytes and adipose invariant natural killer T (iNKT). iNKT cells play a crucial role in dampening obesity-induced white adipose tissue (WAT) inflammation. In particular, adipocytes act as major antigen-presenting cells that regulate the activity and the number of iNKT cells in WAT. However, the regulatory mechanisms that modulate interplay between adipocytes and adipose iNKT cells in obesity are still unclear. **(A)** Function of adipose iNKT cells is influenced by (1) “lipid antigens” loaded on CD1d and (2) “co-stimulatory molecules.” In obesity, a variety of factors can lead to dynamic changes in both types of lipid antigens and combination of costimulatory molecules, resulting in alteration of the functionality of adipose iNKT cells. **(B)** In addition to cytokine production, iNKT cells have the ability to directly or indirectly induce apoptosis. iNKT cells can express fasL and activate NK cells to kill target cells. NK cells activated by IFN-γ secrete perforin/granzyme to promote cell death processes. One of the characteristics of obese WAT is an increase in the number of hypertrophic adipocytes that are susceptible to apoptosis. Therefore, it would be interesting to determine whether adipose iNKT cells can contribute to obesity-induced death of hypertrophic adipocytes in WAT.

White adipose tissue is characterized by a unique immune system that dynamically responds to nutritional stresses. With respect to iNKT cells, WAT provides a special microenvironment that is enriched in profound APCs and diverse activating stimuli (lipid antigens) and cytokines. Although recent reports have demonstrated the distinct characteristics and physiological roles of adipose iNKT cells, the interconnected mechanisms of interplay between adipose iNKT cells and other cells in WAT need to be elucidated (Figure 2). The kinds of endogenous lipid antigens presented by adipocytes and the underlying molecular mechanisms that mediate dynamics of lipid antigen presentation by adipocytes remain to be determined (Figure 2A). Although it has been reported that several regulators of adipocyte differentiation including peroxisome proliferator-activated receptor gamma and CCAAT/enhancer-binding protein (C/EBP)-β and -δ control CD1d expression, the regulatory mechanisms of other necessary machineries involved in lipid antigen synthesis and presentation in adipocytes are still unclear (26, 62). Moreover, it has been proposed that types of co-stimulation can affect iNKT cell functions. For instance, certain types of co-stimulatory molecules, such as CD40/CD40L and CD28/B7.2, are required for the Th1-skewed responses of iNKT cells (63, 64). Therefore, it is worth studying that the dynamics of a repertoire of co-stimulation in WAT in response to nutritional stress in obesity. Finally, even though the major functions of iNKT include cytokine production and cytolytic activity, most studies involving adipose iNKT cells have focused on the contribution of iNKT cell-derived cytokines to adipose tissue inflammation. Given that hypertrophic adipocytes are prone to apoptosis in obese WAT, it would be both interesting and important to investigate the involvement of adipose iNKT cells in the clearance of hypertrophic adipocytes in obesity (Figure 2B).

## Conclusion

The incidence of obesity and metabolic diseases has risen dramatically during the past few decades. Adipose tissue inflammation is considered to be one of the key mechanisms linking obesity and metabolic diseases. A growing body of evidence indicates that the crosstalk between adipocytes and adipose iNKT cells is crucial to regulate WAT homeostasis as well as for adipose tissue inflammation. In this regard, understanding of regulatory mechanisms that modulate interplay between adipocytes and adipose iNKT cells will provide a new approach to control adipose tissue inflammation and metabolic diseases.

## Author Contributions

All authors listed have made a substantial contribution to the work.

## Conflict of Interest Statement

The authors declare that the research was conducted in the absence of any commercial or financial relationships that could be construed as a potential conflict of interest.
